# 2190: Collective capacity building tool (CCBT): A unique instrument and process supporting community-campus partnerships for translation

**DOI:** 10.1017/cts.2017.252

**Published:** 2018-05-10

**Authors:** Kathryn Nearing, Donald Nease, Montelle M. Tamez, Martha Tenney, Elizabeth Sweitzer

## Abstract

OBJECTIVES/SPECIFIC AIMS: (1) Provide an innovative tool used to accelerate and evaluate T3-T4 research; (2) describe the collective capacity building tool (CCBT) methodology—both programmatic and evaluative applications; and (3) share insights about the process and outcomes of community-engaged research. METHODS/STUDY POPULATION: Academic and community-based partners complete the assessment together at the beginning and conclusion of their Community Engagement pilot projects. Further, they are encouraged to use the tool and the associated insights/priorities that emerge as the basis for data-driven coaching with Community Research Liaisons throughout the 12-month grant cycle. RESULTS/ANTICIPATED RESULTS: Pre/post results with 4 cohorts of pilot grantees consistently demonstrated the most positive change in relation to 1 item: overcoming previously identified barriers to community engagement (eg, language, mistrust, scheduling conflicts). Other key findings: (1) networks of reciprocal ties expand, providing structures to support dissemination of information and interventions. (2) Partners leverage expanded networks to pursue follow-on funding and extend the scope/reach of their efforts geographically and/or with new populations. (3) Projects enhance trust in the research process by developing group processes that facilitate the respectful sharing of diverse (often alternative) viewpoints and through culturally-responsive project implementation. DISCUSSION/SIGNIFICANCE OF IMPACT: The CCBT can be used at multiple points in time to help project partners achieve the deliberate integration of CBPR principles in practice and advance community-engaged translational research efforts for sustainability and scalability. The CCBT is sensitive enough to document the iterative nature of partnership development and CBPR. An example: a great deal of variability was found in how formally partners defined roles. Further, partner roles often changed as projects evolved. Still, results indicated a general trend toward achieving greater clarity in partner roles over time. Further, the tool captured set-backs due to partner turn-over and partnerships regaining momentum after new staff came on board. Results have strong face validity: more mature partnerships reported stronger community connections and previous successes to build upon. Perhaps most importantly: the tool and associated process was well-received by academic and community-based partners alike.

